# Functional outcomes in adults with tuberculous meningitis admitted to the ICU: a multicenter cohort study

**DOI:** 10.1186/s13054-018-2140-8

**Published:** 2018-08-17

**Authors:** Marie Cantier, Adeline Morisot, Emmanuel Guérot, Bruno Megarbane, Keyvan Razazi, Damien Contou, Eric Mariotte, Emmanuel Canet, Etienne De Montmollin, Vincent Dubée, Eric Boulet, Stéphane Gaudry, Guillaume Voiriot, Julien Mayaux, Frédéric Pène, Mathilde Neuville, Bruno Mourvillier, Stéphane Ruckly, Lila Bouadma, Michel Wolff, Jean-François Timsit, Romain Sonneville, ENCEPHALITICA study group

**Affiliations:** 1 Department of Intensive Care Medicine and Infectious Diseases, Bichat-Claude Bernard University Hospital, Assistance Publique—Hôpitaux de Paris, 46 rue Henri Huchard, 75018 Paris, France; 2 Department of Public Health, L’Archet Hospital, Nice University Hospital, Nice, France; 3 Department of Intensive Care Medicine, Georges Pompidou European Hospital, Assistance Publique—Hôpitaux de Paris, Paris, France; 4 Department of Intensive Care Medicine and Toxicology, Lariboisière University Hospital, Assistance Publique—Hôpitaux de Paris, Paris, France; 5 Department of Intensive Care Medicine, Henri Mondor University Hospital, Assistance Publique—Hôpitaux de Paris, Paris, France; 6 Department of Intensive Care Medicine, Saint-Louis University Hospital, Assistance Publique—Hôpitaux de Paris, Paris, France; 7 Department of Intensive Care Medicine, Saint-Denis Delafontaine Hospital, Saint-Denis, France; 8 Department of Intensive Care Medicine, Saint-Antoine University Hospital, Assistance Publique—Hôpitaux de Paris, Paris, France; 9 Department of Intensive Care Medicine, René Dubos Hospital, Pontoise, France; 10 Medical-Surgical Intensive Care Unit, Louis Mourier University Hospital, Assistance Publique—Hôpitaux de Paris, Colombes, France; 11 Department of Intensive Care Medicine, Tenon University Hospital, Assistance Publique—Hôpitaux de Paris, Paris, France; 12 Department of Pneumology and Intensive Care Medicine, La Pitié-Salpêtrière University Hospital, Assistance Publique—Hôpitaux de Paris, Paris, France; 13 Department of Intensive Care Medicine, Cochin University Hospital, Assistance Publique—Hôpitaux de Paris, Paris, France; 14 UMR 1137, IAME Team 5, DeSCID: Decision SCiences in Infectious Diseases, control and care, INSERM/Université Paris Diderot, Sorbonne Paris Cité, Paris, France; 15 UMR 1148, Laboratory for Vascular and Translational Science, INSERM/Université Paris Diderot, Sorbonne Paris Cité, Paris, France; 16 Department of Neurology, Saint Antoine University Hospital, Assistance Publique—Hôpitaux de Paris, 184 rue du Faubourg Saint Antoine, 75011 Paris, France

**Keywords:** Tuberculous meningitis, Steroids, Intensive care, Functional outcomes

## Abstract

**Background:**

Tuberculous meningitis (TBM) is a devastating infection in tuberculosis endemic areas with limited access to intensive care. Functional outcomes of severe adult TBM patients admitted to the ICU in nonendemic areas are not known.

**Methods:**

We conducted a retrospective multicenter cohort study (2004–2016) of consecutive TBM patients admitted to 12 ICUs in the Paris area, France. Clinical, biological, and brain magnetic resonance imaging (MRI) findings at admission associated with a poor functional outcome (i.e., a score of 3–6 on the modified Rankin scale (mRS) at 90 days) were identified by logistic regression. Factors associated with 1-year mortality were investigated by Cox proportional hazards modeling.

**Results:**

We studied 90 patients, of whom 61 (68%) had a score on the Glasgow Coma Scale ≤ 10 at presentation and 63 (70%) required invasive mechanical ventilation. Brain MRI revealed infarction and hydrocephalus in 38/75 (51%) and 25/75 (33%) cases, respectively. A poor functional outcome was observed in 55 (61%) patients and was independently associated with older age (adjusted odds ratio (aOR) 1.03, 95% CI 1.0–1.07), cerebrospinal fluid protein level ≥ 2 g/L (aOR 5.31, 95% CI 1.67–16.85), and hydrocephalus on brain MRI (aOR 17.2, 95% CI 2.57–115.14). By contrast, adjunctive steroids were protective (aOR 0.13, 95% CI 0.03–0.56). The multivariable adjusted hazard ratio of adjunctive steroids for 1-year mortality (47%, 95% CI 37%–59%) was 0.23 (95% CI 0.11–0.44). Among survivors at 1 year, functional independence (mRS of 0–2) was observed in 27/37 (73%, 95% CI 59%–87%) cases.

**Conclusions:**

A poor functional outcome in adult TBM patients admitted to the ICU in a nonendemic area is observed in 60% of cases and is independently associated with elevated cerebrospinal fluid protein level and hydrocephalus. Our data also suggest a protective effect of adjunctive steroids, with reduced disability and mortality, irrespective of immune status and severity of disease at presentation. One-year follow-up revealed functional independence in most survivors.

**Electronic supplementary material:**

The online version of this article (10.1186/s13054-018-2140-8) contains supplementary material, which is available to authorized users.

## Background

Tuberculous meningitis (TBM) represents the most severe form of tuberculosis. TBM is a diagnostic and therapeutic challenge in nonendemic areas and may represent an underestimated cause in patients presenting with acute meningoencephalitis [1, 2]. It is frequently associated with neurologic complications requiring admission to the intensive care unit (ICU) [3, 4], including brain infarction, acute hydrocephalus, tuberculomas, and basal arachnoiditis [5].

Diagnosis of TBM remains difficult, mainly based on brain MRI and the isolation of *Mycobacterium tuberculosis* in the CSF, and the initiation of anti-tuberculosis therapy remains empirical [6, 7]. Since 2010, a consensus diagnostic score has been proposed to identify each suspected case as defined, probable, possible, or excluded TBM [8].

Mortality in adult TBM reaches 30–60%, and severe disability is reported in more than 25% of survivors [9, 10]. Main indicators of poor outcome in adult patients include delayed diagnosis, delayed treatment, higher Medical Research Council (MRC) disease severity grade, lower cerebrospinal fluid lymphocyte cell count, and anti-tubercular drug resistance [5, 11, 12]. Adjunctive steroids reduce mortality, but may have no effect on disabling neurologic sequelae in survivors [13, 14]. Moreover, the benefit of steroids is controversial in HIV-infected individuals and in patients with MRC grade 3 illness at presentation [14]. However, most prognostic studies have been conducted in endemic areas of TBM, with no or limited access to intensive care. Data on TBM patients requiring ICU admission are scarce and mainly based on retrospective single-center studies conducted in low or middle-income countries [15–17].

In the present study, we aimed to identify indicators of poor functional outcome in adult patients with severe TBM in a nonendemic area with high access to intensive care. In particular, we investigated the effect of adjunctive steroids on functional outcomes and 1-year mortality.

## Methods

### Study design

We conducted a retrospective cohort study on consecutive adult TBM cases admitted to the medical ICUs of 12 hospitals, located in the Paris area, France, from January 1, 2004 to June 15, 2016. Patients were identified using the national information system (PMSI) with the following ICD-9 codes: lymphocytic meningitis, meningoencephalitis (G049), tuberculous meningoencephalitis (G050, A178), and tuberculous abscess (G07). The ethics committee of the French Society of Intensive Care Medicine (SRLF) approved the study protocol. In accordance with French law, informed consent was not required for this observational study.

### Definitions and inclusion criteria

Patients were included if they fulfilled the diagnosis criteria for TBM, established by the expert consensus score of 2010 [8]. This score is based on clinical information, biological criteria (including CSF analysis), brain imaging, and evidence of tuberculosis elsewhere to classify cases into three TBM categories, based on their total diagnostic score: definite TBM (i.e., microbiological identification or evidence from commercial nucleic acid amplification tests of central nervous system *M. tuberculosis* infection); probable TBM (diagnostic score of 12 or above when imaging is available); and possible TBM (diagnostic score of 6–11 when imaging is available).

Patients were excluded if data on the primary outcome were missing, if an alternative diagnosis was established at hospital discharge, or if a favorable outcome was observed in the absence of anti-tuberculosis therapy.

### Data collection

Standardized methods for enhanced quality and comparability of TBM study guidelines were followed for data collection [18]. Prior health status was assessed by the Knaus score [19]. Patients were considered immunocompromised in the case of HIV infection, solid organ transplantation, solid cancer, hematological malignancy, steroid therapy, chemotherapy, chronic alcoholism, and/or intravenous drug use. A major neurological deficit was defined as monoparesis, hemiparesis, paraparesis, or tetraparesis. Neurological status at admission was staged based on the modified British Medical Research Council (MRC) criteria: stage 1 was defined as a score on the Glasgow Coma Scale (GCS) of 15 and the absence of neurological deficit; stage 2 as a score on the GCS of 11–14, or a score on the GCS of 15 associated with focal neurological sign; and stage 3 as a score on the GCS ≤ 10 [18]. The use of invasive mechanical ventilation, vasopressors, and/or neurosurgical interventions (external ventricular drainage and/or brain biopsy) during the ICU stay was recorded. Bacterial findings were recorded, including types of samples analyzed, microbiological methods used for detection of *M. tuberculosis*, and anti-tuberculosis drug resistance. Initial anti-tuberculosis therapy consisted of a standard regimen with four drugs: isoniazid, rifampicin, ethambutol, and pyrazinamide. In the case of resistance, minor drugs were used [10]. Data on adjunctive steroids at admission (dose, molecule) were collected.

### Outcomes

Functional outcome was graded 90 days after ICU admission according to the modified Rankin Scale (mRS). The primary endpoint was poor functional outcome, defined by a score of 3–6 (i.e., functional dependence, severe disability, or death) on the mRS 90 days after ICU admission. The secondary endpoint was mortality 1 year after ICU admission.

### Statistical analysis

Data are reported as median (interquartile range) or number (%). Patients’ characteristics were compared according to functional outcome at 90 days, using Fisher’s exact tests for categorical variables and Mann–Whitney tests for continuous variables. Durations were calculated from the time of ICU admission. Univariate logistic regression analysis on nonimputed data was performed to evaluate associations between variables and functional outcome. Clinically relevant variables, including use of steroids, and other variables associated with poor outcome in univariate analysis (*p* <  0.1) were included in the multivariate model on imputed data. A stepwise selection was used to select variables to construct the final model. The adjusted odds ratio (aOR) values with 95% confidence intervals (95% CIs) were computed. Univariate Cox proportional hazard modeling on nonimputed data was performed to evaluate associations between variables and 1-year mortality. Clinically relevant variables, including use of steroids, and those associated with mortality in univariate analysis (*p* <  0.1) were entered into the multivariate model on imputed data. Hazard ratios (HRs) and their 95% CIs were computed. Survival rates between patients who received adjunctive steroids and patients who did not were compared with the log-rank test and Kaplan–Meier survival curves were computed. Multiple imputations were performed using the multivariate normal distribution (MVN) method. Sensitivity analysis was conducted to assess the impact of no anti-tuberculosis therapy. All analyses were performed using SAS 9.4 (SAS, Inc.) software. *p* ≤ 0.05 was considered statistically significant.

## Results

### Patients

Among the 112 eligible patients, 22 were excluded (Additional file 1). Baseline characteristics of the 90 included patients (age 43 (29–58) years, 56 (62%) males) are presented in Table 1. Overall, 41 (46%) patients were immunocompromised, including 20 (22%) with HIV infection, and 61 (68%) patients had MRC grade 3 illness at admission. The temperature was 38 (37–39) °C, and fever (*T*° > 38 °C) was documented in 43 (48%) cases. A major neurological deficit was observed in 41 (46%) cases and seizures were noted in 31 (34%) patients (including 12 (13%) with convulsive status epilepticus). Extraneurological findings were reported in 62 (69%) patients.

**Table 1:** Baseline characteristics of patients

Variable	Missing, *n*	All patients (*n* = 90)	Good outcome (*n* = 35)	Poor outcome (*n* = 55)	*p* value
Clinical features					
Age (years)	0	43 (29; 58)	33 (24; 55)	45 (34; 58)	0.04
Male sex	0	56 (62.2)	19 (54.3)	37 (67.3)	0.27
Immunosuppression	0	41 (45.6)	12 (34.3)	29 (52.7)	0.13
Knaus C/D	0	6 (6.7)	1 (2.9)	5 (9.1)	0.40
MRC grade^a^	0				0.06
1		3 (3.3)	2 (5.7)	1 (1.8)	
2		26 (28.9)	14 (40)	12 (21.8)	
3		61 (67.8)	19 (54.3)	42 (76.4)	
GCS score	2	12 (8; 14)	14 (10; 14)	9 (7; 13)	< 0.01
Major focal deficit	0	41 (45.6)	10 (28.6)	31 (56.4)	0.02
Meningeal syndrome	0	55 (61.1)	24 (68.6)	31 (56.4)	0.28
Cranial nerve palsies	0	24 (26.7)	9 (25.7)	15 (27.3)	1.00
Seizures	0	31 (34.4)	9 (25.7)	22 (40)	0.18
Extraneurological signs	0	62 (68.9)	21 (60)	41 (74.5)	0.17
Temperature (°C)	0	38 (37; 39)	38 (37.5; 39)	38 (36.9; 39)	0.53
Laboratory findings					
Serum sodium level (mmol/L)	2	131 (127; 135)	133 (128; 137)	130 (127; 134)	0.35
CSF pleocytosis (cells/μl)^b^	3	130 (27; 300)	140 (36; 350)	120.5 (27; 250)	0.56
CSF lymphocyte (%)^b^	18	73.2 (37; 94)	88.6 (57.1; 96.8)	59 (33.3; 90)	0.04
CSF glucose level (mmol/L)^b^	3	2 (1.1; 3)	2.2 (1.6; 2.6)	1.9 (1; 3.3)	0.71
CSF protein level (g/L)^b^	4	1.9 (1; 3)	1.5 (1.2; 1.9)	2.3 (0.9; 3.5)	0.07
Brain CT Infarction	1	83 (93.3)	32 (94.1)	51 (92.7)	1.00
Infarction	0	12 (14.5)	0 (0)	12 (23.5)	< 0.01
Hydrocephalus	0	16 (19.3)	2 (6.3)	14 (27.5)	0.02
Abscess/tuberculoma	0	19 (22.9)	9 (28.1)	10 (19.6)	0.43
Basal arachnoiditis	0	4 (4.8)	0 (0)	4 (7.8)	0.16
Brain MRI	0	75 (83.3)	29 (82.9)	46 (83.6)	1.00
Infarction	0	38 (50.7)	9 (31)	29 (63)	< 0.01
Hydrocephalus	0	25 (33.3)	4 (13.8)	21 (45.7)	< 0.01
Abscess/tuberculoma	0	35 (46.7)	13 (44.8)	22 (47.8)	0.82
Basal arachnoiditis	0	37 (49.3)	13 (44.8)	24 (52.2)	0.64

Data presented as median (interquartile range) or number (percentage)

*MRC* British Medical Research Council, *GCS* Glasgow Coma Scale, *CSF* cerebrospinal fluid, *CT* computed tomography, *MRI* magnetic resonance imaging

^a^Grade 3 indicates a GCS score ≤ 10

^b^Data available for 87 patients

Hyponatremia was common, with blood sodium levels of 131 (127–135) mmol/L at admission.

CSF analysis revealed a typical pleocytosis of 130 (27–300) cells/μl with a lymphocyte proportion of 73% (37–94%), with elevated CSF protein levels (1.9 (1.0–3.0) g/L) and low glucose levels (2 (1.1–3.0) mmol/L). Bacteriological findings are reported in Additional file 2.

**Fig. 1:**
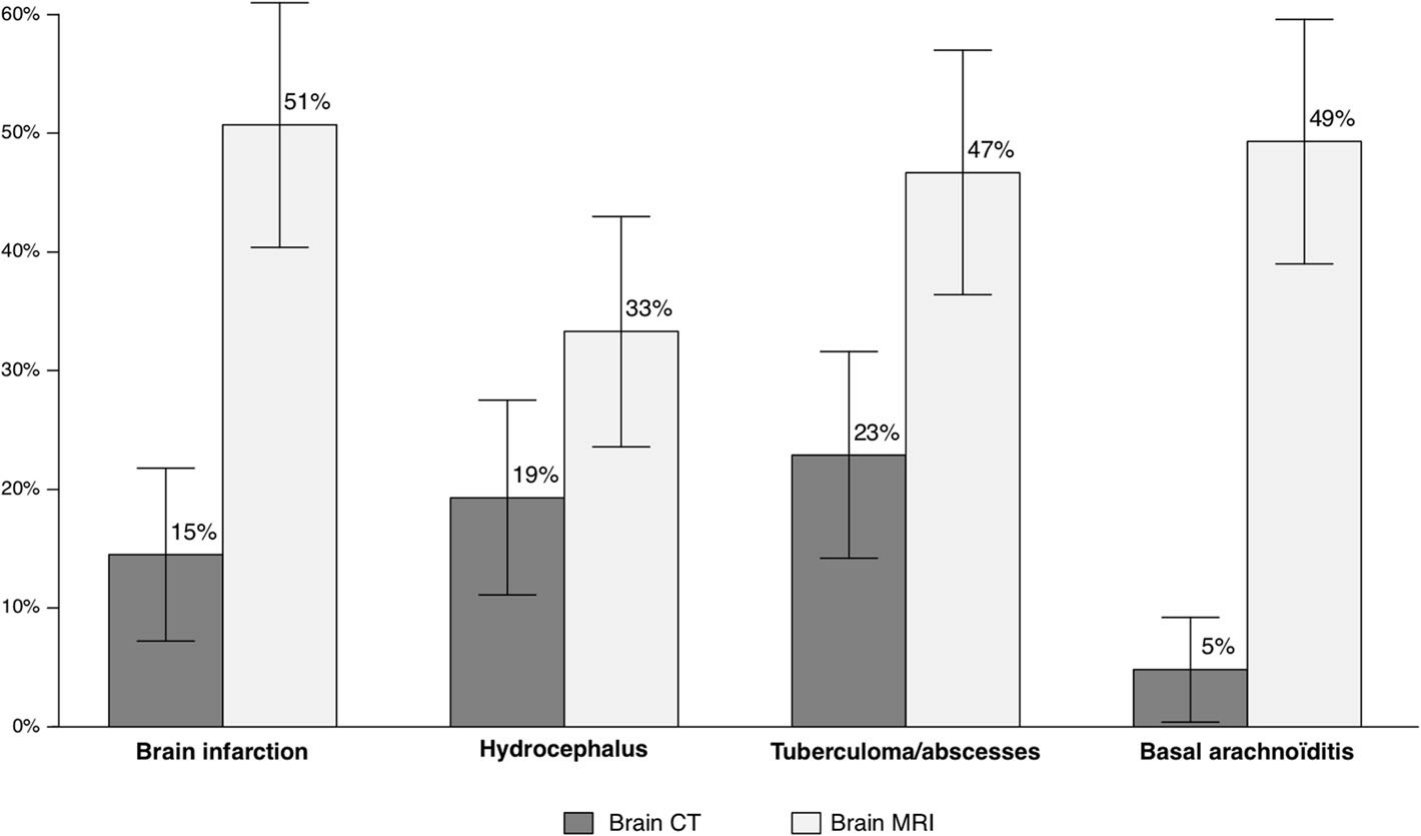
Main neurologic complications detected by brain CT and MRI at admission. Bars indicate percentage with 95% CI. CT computed tomography, MRI magnetic resonance imaging

Brain CT and MRI findings are presented in Table 1. MRI appeared superior to CT for detection of infarction, arachnoiditis, and tuberculoma at ICU admission (Fig. 1).

Based on the consensus diagnostic score, 41 (46%) patients were diagnosed with definite TBM, 28 (31%) patients with probable TBM, and 21 (23%) patients with possible TBM. Data on organ support, specific therapy, and adjunctive steroids are presented in Additional file 3**.**

First-line anti-tuberculosis therapy was initiated in 85 (96%) cases (five patients died before treatment could be started). Anti-tuberculosis drugs for suspected drug-sensitive TBM consisted of a combination of isoniazid (5 mg/kg, maximum 300 mg, 12 months), rifampicin (10 mg/kg, maximum 600 mg, 12 months), ethambutol (15 mg/kg, first 2 months), and pyrazinamide (25 mg/kg, first 2 months). Ten patients were secondarily detected with resistance to standard treatment and switched to other drugs during their ICU stay.

Adjunctive steroids were initiated in 72 (80%) patients. The steroid dose was recorded for 61/72 (93%) patients and was > 0.4 mg/kg/day of dexamethasone equivalent in 15/61 (25%) patients. Overall, a total of 63 (70%) patients required invasive mechanical ventilation, and 36 (40%) patients required vasopressors. Neurosurgery was performed in 18 (20%) patients.

### Outcomes

**Fig. 2:**
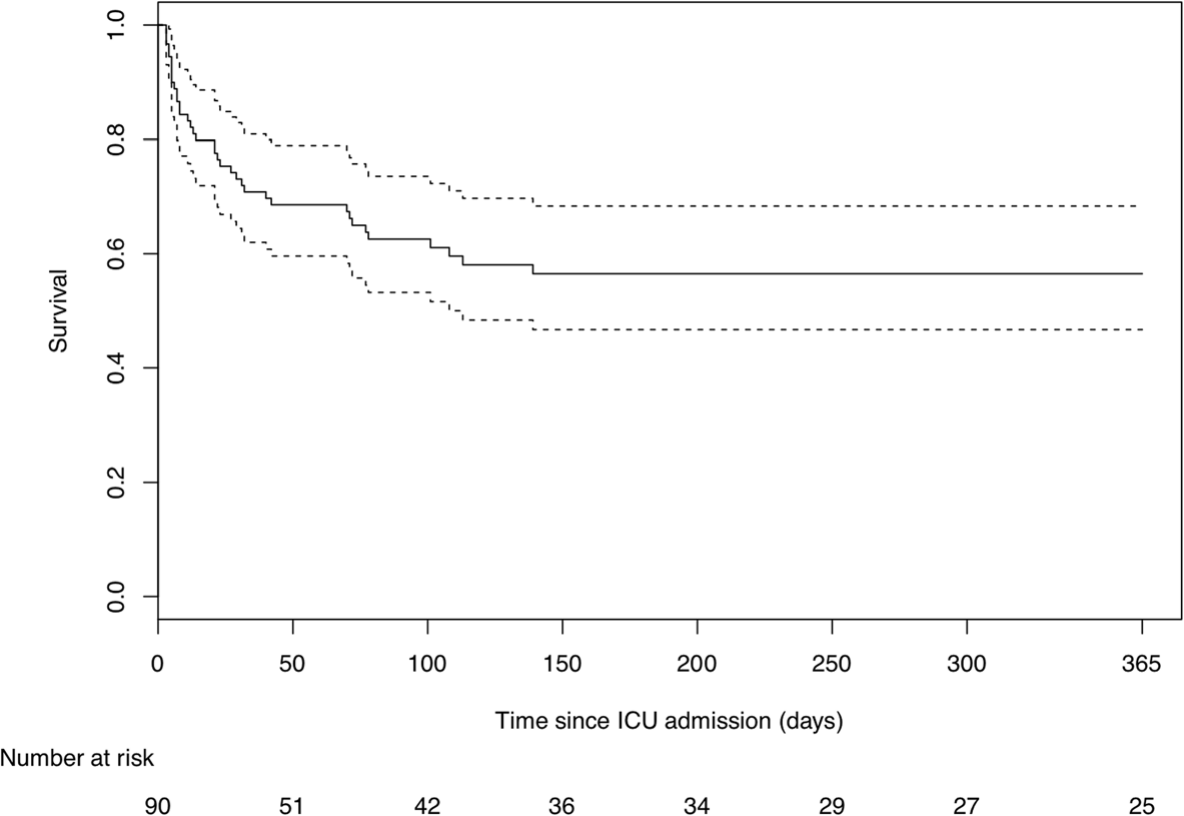
Kaplan–Meier estimates of overall survival at 1 year. ICU intensive care unit

**Fig. 3:**
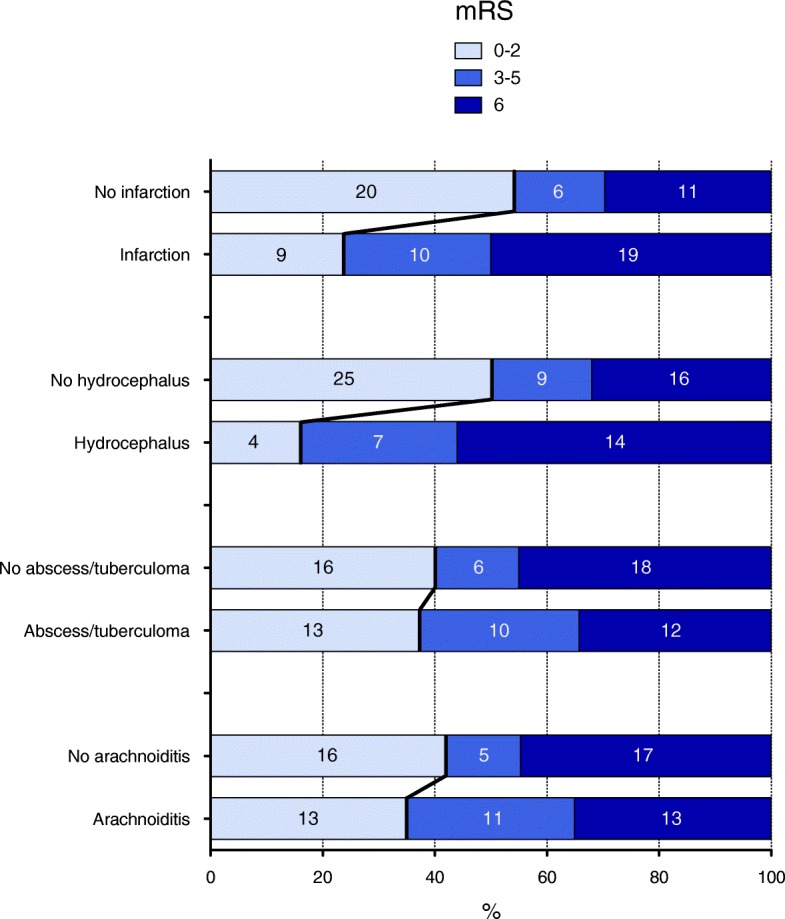
Modified Rankin scale scores at 90 days according to type of neurologic complication. mRS modified Rankin scale

At 90 days, 55/90 (61%) patients had a poor outcome, including 39/90 (43%) deaths (Fig. 2). Most deaths occurred in the ICU (*n* = 36/90, 40%), including 14/36 (39%) because of severe neurological injury. The univariate logistic regression analysis is presented in Additional file 4. Multivariate analysis identified three independent indicators of poor outcome (Table 2): age (aOR 1.03, 95% CI 1.0–1.07), CSF protein level ≥ 2 g/L (aOR 5.31, 95% CI 1.67–16.85), and hydrocephalus on MRI (aOR 17.2, 95% CI 2.57–115.14). By contrast, adjunctive steroids were protective (aOR 0.13, 95% CI 0.03–0.56). Sensitivity analysis conducted after exclusion of the five patients who did not receive anti-tuberculosis therapy displayed no major change in the adjusted odds ratio (aOR 0.22, 95% CI 0.05–1.04). Associations between neurologic complications on brain MRI and outcomes at 90 days are shown in Fig. 3.

**Table 2:** Indicators of poor functional outcome by multivariate logistic regression

Variable	Odds ratio	95% CI	*p* value
Age	1.03	1–1.07	0.04
CSF protein level ≥ 2 g/L	5.31	1.67–16.85	< 0.01
Hydrocephalus on brain MRI			
No MRI	Reference	–	–
No hydrocephalus	1.91	0.45–8.13	0.38
Hydrocephalus	17.2	2.57–115.14	< 0.01
Adjunctive steroids	0.13	0.03–0.56	< 0.01

*CI* confidence interval, *CSF* cerebrospinal fluid, *MRI* magnetic resonance imaging

One-year outcomes were available for 80 patients. Kaplan–Meier analysis estimated the 1-year overall mortality at 47% (95% CI 37–59%) (Fig. 2). Among 1-year survivors, functional independence (mRS of 0–2) was observed in 27/37 (73%, 95% CI 59–87%) cases.

The univariate Cox analysis is presented in Additional file 5. Multivariate Cox regression analysis identified one parameter positively associated with 1-year mortality (Additional file 6): CSF protein level ≥ 2 g/L (aHR 2.22, 95% CI 1.1–4.49). By contrast, adjunctive steroids had an independent protective effect on mortality (aHR 0.23, 95% CI 0.11–0.44). Sensitivity analysis conducted after exclusion of the five patients who did not receive anti-tuberculosis therapy displayed no major change in the adjusted hazard ratio (aHR 0.32, 95% CI 0.14–0.70). Kaplan–Meier survival analysis according to the use of adjunctive steroids is reported in Additional file 7.

An exploratory analysis comparing baseline characteristics and outcomes according to two arbitrarily defined periods is presented in Additional file 8. We observed no major difference in presentation, imaging findings, and outcomes between the two periods.

## Discussion

Our multicenter study, which assessed functional outcomes of adult TBM patients admitted to an ICU in a low tuberculosis prevalence area with high access to intensive care, identified a poor functional outcome in 60% of cases at 90 days. Elevated CSF protein level and hydrocephalus on MRI were independently associated with a poor outcome. Adjunctive steroids were associated with reduced morbidity and mortality, irrespective of immune status or neurologic status at presentation.

Characteristics of patients included in our study differed significantly from those reported in trials conducted in endemic areas with limited access to intensive care [10, 13]. Of note, 68% of patients presented with MRC grade 3 illness, 70% required invasive mechanical ventilation, and brain MRI studies were performed in 83% of patients. Several studies reported a superiority of MRI for diagnosing TBM complications, particularly for the detection of brain infarction in the basal ganglia, brainstem, and posterior fossa [20]. Our data confirm that early MRI evaluation is of paramount importance for detection of complications that may impact intensive care management and help prognostication.

TBM is a devastating disease responsible for severe disability or death in 20–60% of cases [21]. Outcomes of TBM may depend on therapeutic resources, including access to anti-tuberculosis therapy, intensive care facilities, and antiretroviral therapy for HIV-infected patients. In our study, conducted in a nonendemic area with high access to intensive care, 61% of patients had a poor outcome at 90 days. Because of the high mortality rate, functional dependence (i.e., a score on the mRS of 3–5) was observed in only 18% of patients at 90 days and 11% of patients at 1 year. These results contrast with large cohort studies of TBM in Europe and India, reporting higher rates of neurological disability and lower mortality rates [9, 22]. These discrepancies are likely explained by selection bias of the most severe cases requiring ICU admission in our study. Previous data on TBM patients requiring ICU admission are scarce and mainly based on retrospective single-center studies conducted in low or middle-income countries, with more limited diagnostic and therapeutic resources [15–17].

Consistent with previous studies in TBM patients, elevated CSF protein levels were associated with a poor outcome [23, 24]. An elevated protein level was also found to be associated with poor prognosis in other causes of encephalitis, likely reflecting brain damage related to the severity of meningeal inflammation and blood–brain barrier dysfunction [4, 25].

Hydrocephalus is a common complication of TBM that may be observed in up to 65% of patients [26]. In our study, this complication was observed in 33% of cases at admission and was independently associated with a poor outcome, as previously reported [9, 10, 27]. By increasing intracranial pressure, hydrocephalus may be responsible for additional brain injury. Because hydrocephalus in early stages of TBM may resolve completely, emergency therapeutic options (i.e., CSF lumbar or external ventricular drainage) should be systematically proposed [28].

Since 2004, adjunctive steroids are recommended for TBM management of adult patients. In a multicenter randomized controlled trial, dexamethasone was associated with a reduced risk of death at 9 months, without reducing long-term disability [13]. According to a recent Cochrane review, the benefit of steroids remains controversial in patients with MRC grade 3 illness or who are immunocompromised [14]. In our study, which included a high proportion of patients with severe neurologic presentation, steroids were independently associated with reduced morbidity and mortality. These results suggest that steroids are essential in the initial management of patients admitted to the ICU with suspected TBM. Mechanisms of action of steroids in TBM have not been elucidated [5]. Anti-VEGF and anti-inflammatory effects could reduce vasogenic edema and basal meningeal inflammation, preventing intracranial complications such as brain infarction and hydrocephalus. Steroids could also limit the occurrence of adverse events such as liver injury, requiring modification or interruption of anti-tuberculous therapy, associated with poor prognosis [20, 29, 30].

Our study has several strengths, including a multicenter design in a nonendemic area with high access to intensive care. Moreover, we used validated guidelines and consensus definitions for inclusion of patients and reporting of data. Our study has also limitations inherent to its retrospective design. Of note, initial neurological evaluation was not conducted according to a standardized protocol in the different participating centers. However, prospective cohort studies would be difficult to implement, mainly because of the low incidence of TBM in the Paris area. As we focused on the most severe TBM cases admitted to the ICU, our results may not be extrapolatable to a less severe population.

## Conclusions

A poor functional outcome in adult TBM patients admitted to the ICU is observed in 60% of cases and is independently associated with elevated CSF protein level and hydrocephalus. Our data suggest a protective effect of adjunctive steroids, with reduced disability and mortality, irrespective of immune status and severity of disease at presentation.

## Additional files

Additional file 1: **Figure S1.** Flowchart. (DOCX 48 kb)

Additional file 2: **Table S1.** Bacteriological findings. (DOCX 15 kb)

Additional file 3: **Table S2.** Organ support, specific anti-tuberculosis therapy, and adjunctive steroids. (DOCX 16 kb)

Additional file 4: **Table S3.** Univariate logistic regression analysis of factors associated with poor functional outcomes. (DOCX 17 kb)

Additional file 5: **Table S4.** Univariate Cox regression analysis of factors associated with 1-year mortality. (DOCX 17 kb)

Additional file 6: **Table S5.** Cox multivariate analysis of factors associated with mortality. (DOCX 15 kb)

Additional file 7: **Figure S2.** Kaplan–Meier estimates of overall survival at 1 year according to use of adjunctive steroids. (DOCX 27 kb)

Additional file 8: **Table S6.** Patients’ characteristics according to study periods. (DOCX 20 kb)

## Abbreviations

CSF: Cerebrospinal fluid
CT: Computed tomography
GCS: Glasgow Coma Scale
HIV: Human immunodeficiency virus
ICU: Intensive care unit
MRC: Medical Research Council
MRI: Magnetic resonance imaging
mRS: Modified Rankin scale
MVN: Multivariate normal distribution
TBM: Tuberculous meningitis
