# Supplementary material for: Functional outcomes in adults with tuberculous meningitis admitted to the ICU: a multicenter cohort study
Source: Crit Care. 2018 Aug 17;22:210. doi: 10.1186/s13054-018-2140-8 (PMC6098613; doi:10.1186/s13054-018-2140-8)
Supplement: Supplementary file 1 — Figure S1. Flowchart. (DOCX 48 kb) [file 13054_2018_2140_MOESM1_ESM.docx]

**Figure S1. Flowchart**
