# Supplementary material for: Functional outcomes in adults with tuberculous meningitis admitted to the ICU: a multicenter cohort study
Source: Crit Care. 2018 Aug 17;22:210. doi: 10.1186/s13054-018-2140-8 (PMC6098613; doi:10.1186/s13054-018-2140-8)
Supplement: Supplementary file 2 — Table S1. Bacteriological findings. (DOCX 15 kb) [file 13054_2018_2140_MOESM2_ESM.docx]

Table S1. Bacteriological findings

|  | All patients  n=90 |
| --- | --- |
| **Positive sample (AFB or *M. Tuberculosis*)** | 57/90 (63) |
| CSF | 41/90 (46) |
| Pulmonary ^a^ | 29/90 (32) |
| Other ^b^ | 19/90 (21) |
| ≥ 2 types of positive samples | 28/90 (31) |
| **Positive methods for detection** |  |
| Direct examination, Ziehl-Neelsen staining | 20/57 (35) |
| Culture | 36/57 (40) |
| Molecular amplification (PCR) ^c^ | 20/39 (51) |
| PCR + AFB or culture ^d^ | 13/39 (33) |
| **MDR/XDR-TB ^e^**  ^a^ | 10/31 (32) |

Data are numbers (percentages).

Abbreviations: CSF, cerebrospinal fluid; PCR, polymerase chain reaction; AFB, Acid Fast Bacilli; MDR-TB, multidrug-resistant tuberculosis; XDR, extensively drug-resistant tuberculosis.

^a^ Pulmonary samples included sputum and bronchoalveolar lavage.

^b^ Other samples included urines, stool, blood, gastric fluid, and skin biopsy, adenopathy biopsy, brain biopsy, liver biopsy, osteo-medullary biopsy.

^c^ PCR assay for *M. Tuberculosis* was performed in 39/90 patients.

^d^ MDR-TB involves resistance to the two most powerful anti-TB drugs, isoniazid and rifampicin. XDR-TB involves resistance to the two most powerful anti-TB drugs, isoniazid and rifampicin, in addition to resistance to any of the fluoroquinolones (such as levofloxacin or moxifloxacin) and to at least one of the three injectable second-line drugs (amikacin, capreomycin or kanamycin).
