# Supplementary material for: Functional outcomes in adults with tuberculous meningitis admitted to the ICU: a multicenter cohort study
Source: Crit Care. 2018 Aug 17;22:210. doi: 10.1186/s13054-018-2140-8 (PMC6098613; doi:10.1186/s13054-018-2140-8)
Supplement: Supplementary file 3 — Table S2. Organ support, specific anti-tuberculosis therapy, and adjunctive steroids. (DOCX 16 kb) [file 13054_2018_2140_MOESM3_ESM.docx]

**Table S2. Organ support, specific antituberculous therapy and adjunctive steroids.**

| Variables | All patients  n=90 | Good outcome  n=35 | Poor outcome  n=55 | *P* Value |
| --- | --- | --- | --- | --- |
| **Time between first neurological symptoms and ICU admission, days** | 15 (6-30) | 17 (10-25) | 14 (4-35) | 0.37 |
| **Time between ICU admission and anti-infective therapy initiation, days^a^** | 0 (0-3) | 1 (0-3) | 0 (0-1) | 0.04 |
| **Adjunctive steroids** |  |  |  |  |
| Prescription | 72 (80) | 31 (89) | 41 (75) | 0.10 |
| Time between ICU admission and initiation, days^b^ | 0 (0-4) | 1 (0-3) | 0 (0-4) | 0.66 |
| Steroid dose > 0.4 mg/kg/day of dexamethasone equivalent ^c^ | 15/61 (25) | 5/27 (19) | 10/34 (29) | 0.38 |
| **Invasive mechanical ventilation** | 63 (70) | 14 (40) | 49 (89) | <0.001 |
| **Vasopressors** | 36 (40) | 6/35 (17) | 30/55 (55) | <0.001 |
| **Neurosurgery^d^** | 18 (20) | 2/35 (6) | 16/55 (29) | <0.01 |
| **Nosocomial infections^e^** | 28 (30) | 8(23) | 20 (36) | 0.24 |

Data are median (interquartile range) or numbers (percentages)

Abbreviations: ICU, intensive care unit; DXM, dexamethasone

^a^Data were available for 85 patients receiving antituberculous therapy (five patient did not receive antituberculous therapy because of early death within 4 days following ICU admission).

^b^Data were available for 46/72 patients receiving adjunctive steroids.

^c^Data were available for 61/72 patients receiving adjunctive steroids.

^d^Neurosurgery corresponded to extern ventricular drainage (n=16) or surgical resection of a brain lesion (n=2).

^e^Nosocomial infections included ventilator associated pneumonia, catheter infection, bacteremia, urinary tract infection.
