# Supplementary material for: Functional outcomes in adults with tuberculous meningitis admitted to the ICU: a multicenter cohort study
Source: Crit Care. 2018 Aug 17;22:210. doi: 10.1186/s13054-018-2140-8 (PMC6098613; doi:10.1186/s13054-018-2140-8)
Supplement: Supplementary file 4 — Table S3. Univariate logistic regression analysis of factors associated with poor functional outcomes. (DOCX 17 kb) [file 13054_2018_2140_MOESM4_ESM.docx]

Table S3. Univariate logistic regression analysis of factors associated with poor functional outcomes

| Variable | Missing, n | Good outcome  n=35 | Poor outcome  n=55 | OR | 95%CI | P-value |
| --- | --- | --- | --- | --- | --- | --- |
| **Age, yrs** | 0 | 33 [24 ; 55] | 45 [34 ; 58] | 1.024 | [1 ; 1.05] | 0.08 |
| **Male sex** | 0 | 19 (54.3) | 37 (67.3) | 1.731 | [0.72 ; 4.14] | 0.22 |
| **Immunosuppression** | 0 | 12 (34.3) | 29 (52.7) | 2.138 | [0.89 ; 5.13] | 0.09 |
| **MRC grade 3**^a^ | 0 | 19 (54.3) | 42 (76.4) | 2.720 | [1.09 ; 6.76] | 0.03 |
| **CSF protein level ≥ 2 g / L** | 4 | 9 (27.3) | 32 (60.4) | 4.063 | [1.58 ; 10.44] | <.01 |
| **Brain infarction on MRI** |  |  |  |  |  |  |
| Brain infarction | 0 | 9 (25.7) | 29 (52.7) | 2.148 | [0.6 ; 7.69] | 0.24 |
| No brain infarction | 0 | 20 (57.1) | 17 (30.9) | 0.567 | [0.17 ; 1.92] | 0.36 |
| No MRI | 0 | 6 (17.1) | 9 (16.4) | 1.000 |  | 0.03 |
| **Hydrocephalus on MRI** |  |  |  |  |  |  |
| Hydrocephalus | 0 | 4 (11.4) | 21 (38.2) | 3.500 | [0.79 ; 15.48] | 0.10 |
| No hydrocephalus | 0 | 25 (71.4) | 25 (45.5) | 0.667 | [0.21 ; 2.15] | 0.50 |
| No MRI | 0 | 6 (17.1) | 9 (16.4) | 1.000 |  | 0.03 |
| **Brain abscess on MRI** |  |  |  |  |  |  |
| Brain abscess | 0 | 13 (37.1) | 22 (40) | 1.128 | [0.33 ; 3.9] | 0.85 |
| No brain abscess | 0 | 16 (45.7) | 24 (43.6) | 1.000 | [0.3 ; 3.36] | 1.00 |
| No MRI | 0 | 6 (17.1) | 9 (16.4) | 1.000 |  | 0.96 |
| **Basal arachnoiditis on MRI** |  |  |  |  |  |  |
| Basal arachnoiditis | 0 | 13 (37.1) | 24 (43.6) | 1.231 | [0.36 ; 4.23] | 0.74 |
| No basal arachnoiditis | 0 | 16 (45.7) | 22 (40) | 0.917 | [0.27 ; 3.1] | 0.89 |
| No MRI | 0 | 6 (17.1) | 9 (16.4) | 1.000 |  | 0.82 |
| **Adjunctive steroids** | 0 | 31 (88.6) | 41 (74.5) | 0.378 | [0.11 ; 1.26] | 0.11 |

Data are median (interquartile range) or numbers (percentages)

Abbreviations: MRC: British Medical Research Council; CSF: cerebrospinal fluid.

^a^ A grade 3 on the MRC indicates a Glasgow coma score ≤10.
