# Supplementary material for: Functional outcomes in adults with tuberculous meningitis admitted to the ICU: a multicenter cohort study
Source: Crit Care. 2018 Aug 17;22:210. doi: 10.1186/s13054-018-2140-8 (PMC6098613; doi:10.1186/s13054-018-2140-8)
Supplement: Supplementary file 5 — Table S4. Univariate Cox regression analysis of factors associated with 1-year mortality. (DOCX 17 kb) [file 13054_2018_2140_MOESM5_ESM.docx]

Table S4. Univariate Cox regression analysis of factors associated with 1-year mortality

| Variables | Missing, n | Alive at 1 year  n=53 | Dead at 1 year  n=37 | HR | 95%CI | P-value |
| --- | --- | --- | --- | --- | --- | --- |
| **Age, yrs** | 0 | 38 [28; 58] | 44 [34; 57] | 1.011 | [0.99-1.03] | 0.2741 |
| **Male sex** | 0 | 29 (54.7) | 27 (73) | 1.947 | [0.94-4.04] | 0.0734 |
| **Immunosuppression** | 0 | 19 (35.8) | 22 (59.5) | 1.996 | [1.03-3.85] | 0.0394 |
| **MRC grade 3** | 0 | 34 (64.2) | 27 (73) | 1.339 | [0.65-2.77] | 0.4310 |
| **CSF protein level ≥ 2 g / L** | 4 | 18 (35.3) | 23 (65.7) | 2.375 | [1.18-4.79] | 0.0157 |
| **Brain infarction on MRI** |  |  |  |  |  |  |
| Brain infarction | 0 | 20 (37.7) | 18 (48.6) | 0.588 | [0.26-1.31] | 0.1946 |
| No brain infarction | 0 | 27 (50.9) | 10 (27) | 0.310 | [0.13-0.77] | 0.0113 |
| No MRI | 0 | 6 (11.3) | 9 (24.3) | . |  | 0.0389 |
| **Hydrocephalus on MRI** |  |  |  |  |  |  |
| Hydrocephalus | 0 | 13 (24.5) | 12 (32.4) | 0.641 | [0.27-1.52] | 0.3142 |
| No hydrocephalus | 0 | 34 (64.2) | 16 (43.2) | 0.362 | [0.16-0.82] | 0.0154 |
| No MRI | 0 | 6 (11.3) | 9 (24.3) | . |  | 0.0449 |
| **Brain abscess on MRI** |  |  |  |  |  |  |
| Brain abscess | 0 | 24 (45.3) | 11 (29.7) | 0.351 | [0.14-0.85] | 0.0206 |
| No brain abscess | 0 | 23 (43.4) | 17 (45.9) | 0.539 | [0.24-1.21] | 0.1353 |
| No MRI | 0 | 6 (11.3) | 9 (24.3) | . |  | 0.0681 |
| **Basal arachnoiditis on MRI** |  |  |  |  |  |  |
| Basal arachnoiditis | 0 | 25 (47.2) | 12 (32.4) | 0.374 | [0.16-0.89] | 0.0264 |
| No basal arachnoiditis | 0 | 22 (41.5) | 16 (43.2) | 0.522 | [0.23-1.19] | 0.1203 |
| No MRI | 0 | 6 (11.3) | 9 (24.3) | . |  | 0.0822 |
| **Adjunctive steroids** | 0 | 49 (92.5) | 23 (62.2) | 0.220 | [0.11-0.43] | <.0001 |

Data are median (interquartile range) or numbers (percentages)

Abbreviations: MRC: British Medical Research Council; CSF: cerebrospinal fluid.
