# Supplementary material for: Functional outcomes in adults with tuberculous meningitis admitted to the ICU: a multicenter cohort study
Source: Crit Care. 2018 Aug 17;22:210. doi: 10.1186/s13054-018-2140-8 (PMC6098613; doi:10.1186/s13054-018-2140-8)
Supplement: Supplementary file 6 — Table S5. Cox multivariate analysis of factors associated with mortality. (DOCX 15 kb) [file 13054_2018_2140_MOESM6_ESM.docx]

#### Table S5. Cox multivariate analysis of factors associated with mortality

| Variables | Hazard Ratio | 95% CI | *P* value |
| --- | --- | --- | --- |
| **CSF protein level ≥ 2 g / L** | 2.22 | 1.1-4.49 | 0.03 |
| **Adjunctive steroids** | 0.23 | 0.11-0.44 | < 0.01 |

Abbreviations: CI: confidence interval; MRC: British Medical Research Council; CSF: cerebrospinal fluid.
