# Supplementary material for: Functional outcomes in adults with tuberculous meningitis admitted to the ICU: a multicenter cohort study
Source: Crit Care. 2018 Aug 17;22:210. doi: 10.1186/s13054-018-2140-8 (PMC6098613; doi:10.1186/s13054-018-2140-8)
Supplement: Supplementary file 8 — Table S6. Patients’ characteristics according to study periods. (DOCX 20 kb) [file 13054_2018_2140_MOESM8_ESM.docx]

**Table S6. Patients’ characteristics according to study periods**

| Characteristic | miss | 2004-2009  n=39 | 2010-2016  n=51 | *P* Value |
| --- | --- | --- | --- | --- |
| **Clinical features** |  |  |  |  |
| Age, years | 0 | 42 [30 ; 57] | 43 [26 ; 58] | 0.79 |
| Male sex | 0 | 26 (66.7) | 30 (58.8) | 0.51 |
| Immunosuppression | 0 | 18 (46.2) | 23 (45.1) | 1.00 |
| Knaus C/D | 0 | 2 (5.1) | 4 (7.8) | 0.69 |
| MRC grade | 0 |  |  | 0.92 |
| 1 |  | 1 (2.6) | 2 (3.9) |  |
| 2 |  | 12 (30.8) | 14 (27.5) |  |
| 3 |  | 26 (66.7) | 35 (68.6) |  |
| GCS | 2 | 12 [8 ; 14] | 10 [7 ; 14] | 0.29 |
| Major focal deficit | 0 | 16 (41) | 25 (49) | 0.52 |
| Meningeal syndrome | 0 | 25 (64.1) | 30 (58.8) | 0.67 |
| Cranial nerve palsies | 0 | 10 (25.6) | 14 (27.5) | 1.00 |
| Seizures | 0 | 14 (35.9) | 17 (33.3) | 0.83 |
| Extra-neurological symptoms | 0 | 25 (64.1) | 37 (72.5) | 0.49 |
| Temperature, °C | 0 | 38 [37 ; 38.7] | 38 [36.9 ; 39] | 0.98 |
| **Laboratory findings** |  |  |  |  |
| Serum sodium level, mmol/L | 2 | 129 [126 ; 133] | 133 [129 ; 138] | <.01 |
| CSF pleocytosis, cells/μL | 3 | 172.5 [64 ; 350] | 90 [17 ; 220] | 0.02 |
| CSF lymphocyte proportion, % | 18 | 64.6 [34.2 ; 94] | 77 [47.1 ; 94] | 0.66 |
| CSF glucose level, mmol/L | 3 | 2 [1.1 ; 2.8] | 2 [1.2 ; 3.1] | 0.78 |
| CSF protein level, g/L | 4 | 2.2 [1.4 ; 3.2] | 1.6 [0.8 ; 2.8] | 0.14 |
| **Brain CT**  Infarction | 1 | 36 (94.7) | 47 (92.2) | 1.00 |
| Infarction | 0 | 6 (16.7) | 6 (12.8) | 0.76 |
| Hydrocephalus | 0 | 11 (30.6) | 5 (10.6) | 0.03 |
| Abscesses/tuberculomas | 0 | 8 (22.2) | 11 (23.4) | 1.00 |
| Basal arachnoiditis | 0 | 4 (11.1) | 0 (0) | 0.03 |
| **Brain MRI** | 0 | 30 (76.9) | 45 (88.2) | 0.17 |
| Infarction | 0 | 15 (50) | 23 (51.1) | 1.00 |
| Hydrocephalus | 0 | 11 (36.7) | 14 (31.1) | 0.63 |
| Abscesses/tuberculomas | 0 | 13 (43.3) | 22 (48.9) | 0.81 |
| Basal arachnoiditis | 0 | 14 (46.7) | 23 (51.1) | 0.81 |
| **Poor outcome** | 0 | 24 (61.5) | 31 (60.8) | 1.00 |
